# Production of SARS-CoV-2 N Protein-Specific Monoclonal Antibody and Its Application in an ELISA-Based Detection System and Targeting the Interaction Between the Spike C-Terminal Domain and N Protein

**DOI:** 10.3389/fmicb.2021.726231

**Published:** 2021-12-07

**Authors:** Dongbum Kim, Jinsoo Kim, Sangkyu Park, Minyoung Kim, Kyeongbin Baek, Mijeong Kang, Jun-Kyu Choi, Sony Maharjan, Madhav Akauliya, Younghee Lee, Hyung-Joo Kwon

**Affiliations:** ^1^Institute of Medical Science, College of Medicine, Hallym University, Chuncheon, South Korea; ^2^Department of Microbiology, College of Medicine, Hallym University, Chuncheon, South Korea; ^3^Department of Biochemistry, College of Natural Sciences, Chungbuk National University, Cheongju, South Korea

**Keywords:** SARS-CoV-2, spike protein, N protein, anti-SARS-CoV-2 N antibody, bait and prey, virus detection, ELISA

## Abstract

SARS-CoV-2 infections continue to spread quickly by human-to-human transmission around the world. Therefore, developing methods to rapidly detect SARS-CoV-2 with high sensitivity are still urgently needed. We produced a monoclonal antibody that specifically detects the N protein of SARS-CoV-2 and recognizes N protein in cell lysates of SARS-CoV-2–infected Vero cells but not in cell lysates of MERS-CoV- or HCoV-OC43-infected Vero cells. This antibody recognized N protein in SARS-CoV-2 clades S, GR, and GH and recognized N protein in all the SARS-CoV-2 clades to ∼300 pfu. Previously, we reported that the coronavirus N protein interacts with the C-terminal domain of the spike protein (Spike CD). In this study, we developed an ELISA-based “bait and prey” system to confirm the interaction between SARS-CoV-2 Spike CD and N protein using recombinant fusion proteins. Furthermore, this system can be modified to quantitatively detect SARS-CoV-2 in culture media of infected cells by monitoring the interaction between the recombinant Spike CD fusion protein and the viral N protein, which is captured by the N protein–specific antibody. Therefore, we conclude that our N protein–specific monoclonal antibody and our ELISA-based bait and prey system could be used to diagnose SARS-CoV-2 infections.

## Introduction

Coronaviruses are in the family Coronaviridae and contain genomes composed of positive-sense single-stranded RNA. Coronaviruses generally cause mild, common respiratory infections. However, recently, severe acute respiratory syndrome coronavirus (SARS-CoV), Middle East respiratory syndrome coronavirus (MERS-CoV), and severe acute respiratory syndrome coronavirus 2 (SARS-CoV-2) have caused lethal endemics and pandemics in humans. Like SARS-CoV and MERS-CoV, SARS-CoV-2 belongs to the betacoronavirus genus and has a ∼30-kb genome containing genes that encode for structural spike (S), nucleocapsid (N), envelope, and membrane proteins (Khailany et al., 2020).

Since the outbreak of coronavirus disease 2019 (COVID-19), caused by SARS-CoV-2 infection, was first reported in December 2019 (Wu and McGoogan, 2020; Zhou P. et al., 2020), the COVID-19 pandemic continues throughout the world, despite the recent start of vaccine administration (WHO, 2021). SARS-CoV-2 has been spreading quickly among humans, including through symptomatic, pre-symptomatic, asymptomatic, and environmental transmission (Ferretti et al., 2020). It has been suggested that host and viral determinants significantly influence SARS-CoV-2 transmission efficiency, and researchers are actively working to understand the dynamics of its transmission (Chu et al., 2021). Currently, quantitative real-time reverse transcription PCR (qRT-PCR) is used worldwide to diagnose COVID-19 patients, followed by quarantine of positive patients. Although qRT-PCR is sensitive and is the most specific method for diagnosing COVID-19, this method does not provide rapid results and requires specialized facilities, equipment, and well-trained personnel.

To overcome the limitations of molecular diagnosis, immunological diagnosis is also performed to detect SARS-CoV-2 structural proteins, including S and N proteins. Detection of SARS-CoV-2 using antibodies against its S protein has been developed for rapid diagnosis (Kavithaa et al., 2020; Zheng et al., 2020). Recently, a method for rapid detection of SARS-CoV-2 S protein using the SARS-CoV-2 human angiotensin converting enzyme 2 (ACE2) receptor has been proposed (Lee et al., 2021). Currently, most studies are being performed to detect SARS-CoV-2 using antibodies against SARS-CoV-2 N protein. For example, the COVID-19 Ag Respi-Strip assay (Coris BioConcept, Gembloux, Belgium) detects SARS-CoV-2 using antibodies against SARS-CoV-2 N protein (Mertens et al., 2020; Hodge et al., 2021).

Previously, we suggested a direct interaction between the C-terminal domain (CD) of SARS-CoV-2 S protein (SARS-CoV-2 Spike CD) and the N protein of SARS-CoV-2 (Park et al., 2021a). In this study, we produced a monoclonal antibody against the N protein of SARS-CoV-2 by immunizing mice with a complex of SARS-CoV-2 N protein co-encapsulated with CpG-DNA in a phosphatidyl-β-oleoyl-γ-palmitoyl ethanolamine (DOPE):cholesterol hemisuccinate (CHEMS) complex as described previously (Kim et al., 2011). We also evaluated whether a fusion protein containing SARS-CoV-2 Spike CD and the Fc domain (SARS-CoV-2 Spike CD-Fc) could recognize SARS-CoV-2 N protein. Further, we suggest this approach as a SARS-CoV-2 detection method, which uses the SARS-CoV-2 Spike CD-Fc fusion protein and the anti-SARS-CoV-2 N protein-specific monoclonal antibody.

## Materials and Methods

### Cell Culture and Virus

African green monkey kidney Vero cells and human airway epithelial Calu-3 cells were purchased from the Korean Cell Line Bank (Seoul, South Korea). The cells were maintained in Dulbecco’s modified Eagle’s medium (DMEM; Thermo Fisher Scientific, Waltham, MA, United States) containing 10% fetal bovine serum (FBS; Thermo Fisher Scientific), 25 mM HEPES, 100 U/ml penicillin, and 100 μg/ml streptomycin in a 5% CO_2_ incubator at 37^°^C. MERS-CoV (MERS-CoV/KOR/KNIH/002_05_2015), SARS-CoV-2 S clade (BetaCoV/Korea/KCD03/2020, NCCP43326), GR clade (hCoV-19/Korea/KDCA51463/2021, NCCP43381), and GH clade (hCoV-19/Korea/KDCA55905/2021, NCCP43382) were provided by the National Culture Collection for Pathogens (Osong, South Korea). HCoV-OC43 (KBPV-VR-8) was obtained from the Korea Bank for Pathogenic Viruses (College of Medicine, Korea University, Seoul, South Korea). Virus amplification was performed as described previously (Park et al., 2019, 2021a,b). Briefly, 2 × 10^5^ Vero cells (six-well plates; Corning, NY, United States) were cultured in DMEM containing 10% FBS at 37^°^C in a CO_2_ incubator overnight. After washing with phosphate-buffered saline (PBS), SARS-CoV-2 in PBS at a multiplicity of infection (MOI) of 0.01 was inoculated into each well and then incubated for 1 h in a 5% CO_2_ incubator at 37°C with shaking every 15 min. After 1 h, cell culture medium was replaced with DMEM containing 2% FBS (2 ml) and cells were cultured for 72 h at 37^°^C in a CO_2_ incubator. Cell culture supernatants were harvested and centrifuged for 10 min at 3,000 rpm to remove cell debris. The cleared supernatants were collected and then virus titers were quantified by plaque assay as described previously (Kandeel et al., 2020; Kim et al., 2021). The virus containing media was stored at −70^°^C. MERS-CoV and SARS-CoV-2 amplification and cell culture procedures were performed according to biosafety level 3 (BSL-3) conditions in the Hallym Clinical and Translational Science Institute in accordance with the recommendations of the Institutional Biosafety Committee of Hallym University. HCoV-OC43 amplification and cell culture procedures were performed according to biosafety level 2 (BSL-2) conditions.

### Plaque Assays

Vero cells (6 × 10^5^ cells/well) were cultured on six-well plates in DMEM containing 10% FBS at 37^°^C in a CO_2_ incubator for 18 h. The cells were washed with PBS and infected with 10-fold serial dilutions of each MERS- CoV-, SARS-CoV- 2-, or HCoV-OC43-infected culture supernatants for 1 h in a 5% CO_2_ incubator at 37°C with shaking every 15 min. After removing the medium, 3 ml DMEM/F12 medium (Thermo Fisher Scientific) mixed with 2% Oxoid agar was added to the wells. At 3 days post-infection for MERS-CoV (Park et al., 2019) and SARS-CoV-2 (Park et al., 2021b), and 5 days post-infection for HCoV-OC43 (Maharjan et al., 2021), the overlay medium was removed, and the cells were stained with 0.1% crystal violet for 1 h and washed to count plaques.

### Construction and Expression of Biotin Peptide-6 × His-Tagged Coronavirus N Proteins

To obtain recombinant biotin peptide and 6 *×* His-tagged coronavirus (MERS-CoV and SARS-CoV-2) N proteins (recombinant SARS-CoV-2 N-Bio-His_6_ protein and recombinant MERS-CoV N-Bio-His_6_ protein), the nucleotide sequences coding for SARS-CoV-2 (or MERS-CoV) N protein and biotin peptide (NSGSLHHILDAQKMVWNHR) and 6 *×* His (DRNLPPLAPLGPHHHHHH) fusion were synthesized and cloned. The biotin peptide sequence is recognized by *Escherichia coli* biotin holoenzyme synthetase BirA (Schatz, 1993; Altman et al., 1996; Brown et al., 1998). The nucleotide sequences for the N proteins were retrieved from GenBank: MN908947.3 (nucleotide numbers 28274–29530) for SARS-CoV-2 N protein and KT029139.1 (nucleotide numbers 28566–29804) for MERS-CoV N protein. The nucleotide sequences coding for SARS-CoV-2 N protein-Bio-His_6_ and MERS-CoV N protein-Bio-His_6_ fusions were synthesized (Bioneer, South Korea) with *Not*I and *Kpn*I restriction sites at the 5′ and 3′ ends, respectively. The fusions were inserted into a modified pcDNA 3.4 expression vector (Thermo Fisher Scientific) containing IL-2 signal sequences (pcDNA3.4-MERS-CoV N-Bio-His_6_ and pcDNA3.4-SARS-CoV-2 N-Bio-His_6_) for mammalian cell expression. The recombinant MERS-CoV N-Bio-His_6_ and SARS-CoV-2 N-Bio-His_6_ proteins were expressed in Chinese hamster ovary (CHO) cells harboring an expression vector containing *E. coli* BirA (Catalog No. 32408; Addgene, Watertown, MA, United States) using the Gibco ExpiCHO Expression System Kit (Catalog No. A29133; Thermo Fisher Scientific). To obtain recombinant proteins without biotinylation (SARS-CoV-2 N-His_6_), recombinant MERS-CoV N-Bio-His_6_ and SARS-CoV-2 N-Bio-His_6_ proteins were expressed in cells without the BirA vector. After 14 days of cell culture at 32^°^C, recombinant proteins were purified from cell culture supernatants using Ni-NTA agarose (Qiagen, Hilden, Germany) chromatography and size-exclusion gel chromatography. Expression of recombinant proteins was confirmed by western blot analysis with anti-His-tag antibody (Catalog No. MA1-21315; Thermo Fisher Scientific) and peroxidase-conjugated streptavidin (Catalog No. S5512; Sigma-Aldrich, St. Louis, MO, United States).

### Construction and Expression of Coronavirus Spike CD-Human Fc Fusion Proteins

Fusions of SARS-CoV-2 Spike C-terminal domain (CD) (SARS-CoV-2 Spike CD, GenBank ID: MN908947.3, nucleotide number. 25262–25381; protein QHD43416.1, amino acid number 1234–1273) and human IgG1 Fc domain (GenBank ID: AK123800.1), and MERS-CoV Spike CD (GenBank: KT029139.1, nucleotide number 25416–25514; protein AKL59401.1, amino acid number 1321–1353) and human IgG1 Fc domain were synthesized (Bioneer, South Korea) with *Not*I and *Kpn*I restriction sites at the 5′ and 3′ ends, respectively. The synthesized fusions were inserted into a modified pcDNA 3.4 expression vector (Invitrogen, Waltham, MA, United States) containing IL-2 signal sequences (pcDNA3.4-SARS-CoV-2 Spike CD-Fc, pcDNA3.4-MERS-CoV Spike CD-Fc) for mammalian cell expression using the Gibco ExpiCHO Expression System Kit. Each coronavirus CD-human Fc fusion protein (MERS-CoV Spike CD-Fc, SARS-CoV-2 Spike CD-Fc) was purified from ExpiCHO culture supernatants after 14 days of cell culture at 32^°^C using Protein A affinity chromatography. Expression of recombinant MERS-CoV Spike CD-Fc and SARS-CoV-2 Spike CD-Fc proteins was confirmed by western blot analysis with anti-human IgG Fc antibody (Catalog No. 790-035-098; Jackson ImmunoResearch Laboratories, PA, United States).

### Mice Immunization

BALB/c (4-week-old, female, H-2*^b^*) mice were purchased from Nara-Biotec (Seoul, South Korea). Recombinant SARS-CoV-2 N-Bio-His_6_ protein (50 μg) and CpG-DNA (50 μg) were combined with the DOPE:CHEMS complex (molar ratio of 1:1) as described previously (Kim et al., 2011). The SARS-CoV-2 N protein complex was injected into BALB/c mice intraperitoneally (i.p.) three times at 10-day intervals. Mouse sera were collected by orbital bleeding at 10 days after final injection and then titers of total IgG were measured in 96-well immunoplates coated with recombinant SARS-CoV-2 N-Bio-His_6_ protein by standard ELISA as described previously (Kim et al., 2011). Animal experiments were approved by the Institutional Animal Care and Use Committee of Hallym University (HallymR12020-26, Hallym2021-12).

### Production of Mouse Monoclonal Antibody Against SARS-CoV-2 N Protein

Splenocytes from recombinant SARS-CoV-2 N-Bio-His_6_ protein-immunized mice were fused with mouse SP2/0 myeloma cells in a polyethylene glycol solution (PEG; Sigma-Aldrich). After fusion, hybridoma cells were cloned in HAT medium (Sigma-Aldrich) and HT medium (Sigma-Aldrich) according to the standard hybridoma production method described previously (Kim et al., 2011). The cloned hybridoma cells were injected into the peritoneal cavity of BALB/c mice and then ascites containing the monoclonal antibody were collected. Monoclonal antibody against SARS-CoV-2 N protein (clone 1G10C4 mAb) was purified from the ascitic fluid using Protein A column chromatography.

### Antigen-Specific Ig ELISA

Immunoplates (96-well; Thermo Fisher Scientific) were coated with streptavidin (2 μg/well) overnight in ELISA coating buffer (0.1 M carbonate buffer, pH 9.6) at 4^°^C and then blocked with PBS supplemented with 0.1% Tween-20 (PBST) containing 3% bovine serum albumin (BSA). Recombinant SARS-CoV-2 N-Bio-His_6_ protein (3 μg/well) was added to each well to measure SARS-CoV-2 N protein-specific antibody levels in mouse sera, hybridoma culture supernatants, ascites, and purified monoclonal antibody solution by standard ELISA as described previously (Kim et al., 2011). To identify the subclass of the monoclonal antibody, horseradish peroxidase (HRP)-conjugated anti-mouse IgG (each subclass) antibodies (Southern Biotech, Birmingham, AL, United States) were used. The sensitivity of the SARS-CoV-2 N protein-specific monoclonal antibody (clone 1G10C4 mAb) was measured by ELISA. Then 96-well immunoplates were coated with streptavidin (2 μg/well) overnight in the ELISA coating buffer at 4^°^C and then blocked with PBST containing 3% BSA (blocking buffer). Serially diluted recombinant SARS-CoV-2 N-Bio-His_6_ protein in 100 μl blocking buffer was added to each well and incubated for 2 h. After washing with PBST, monoclonal antibody (3 μg/well, clone 1G10C4 mAb) in PBST was added to each well. After incubation for 2 h at room temperature, HRP-conjugated anti-mouse IgG antibody (1:5,000 dilution, Catalog No. 715-035-150; Jackson ImmunoResearch Laboratories) was added to each well. After washing with PBST, tetramethylbenzidine (TMB) peroxidase substrate (Kirkegaard & Perry Laboratories, Gaithersburg, MD, United States) was added to each well, and then the absorbance at 450 nm of each well was measured using the Spectra Max 250 microplate reader (Molecular Devices, San Jose, CA, United States).

### Measurement of Monoclonal Antibody Binding Affinity by ELISA

The binding affinity of the SARS-CoV-2 N protein-specific monoclonal antibody (clone 1G10C4 mAb) was measured by ELISA as described previously (Park et al., 2019). Briefly, 96-well immunoplates were coated with streptavidin (2 μg/well) overnight in the ELISA coating buffer and then treated with blocking buffer. Recombinant SARS-CoV-2 N-Bio-His_6_ protein (3 μg/well) was added into each well and incubated for 2 h at room temperature. After washing with PBST, serially diluted (1:5) monoclonal antibody (clone 1G10C4 mAb) in PBST was added to each well. After incubation for 2 h at room temperature, HRP-conjugated anti-mouse IgG antibody (1:5,000 dilution) was added to each well. After washing with PBST, TMB peroxidase substrate was added to each well, and then the absorbance at 450 nm of each well was measured using the Spectra Max 250 microplate reader. SigmaPlot was used to determine the EC50 value as described previously (Park et al., 2019).

### Preparation of Virus-Infected Cell Lysates

To determine the specificity of SARS-CoV-2 N protein-specific monoclonal antibody (clone 1G10C4 mAb), cell lysates were prepared from SARS-CoV- 2-, MERS- CoV-, or HCoV-OC43-infected Vero cells. Vero cells (3 × 10^5^ cells) were cultured in six-well plates for 18 h. The cells were washed with PBS and then each virus in PBS at an MOI of 0.1 was inoculated into each well and then incubated for 1 h in a 5% CO_2_ incubator at 37°C with shaking every 15 min. The cells were washed with PBS, and then cultured in 2 ml of DMEM/F12 medium for MERS-CoV or DMEM medium containing 2% FBS for SARS-CoV-2 and HCoV-OC43 at 37^°^C in a CO_2_ incubator. After 72 h, the cells were washed with PBS and then were lysed for 30 min with cell lysis buffer containing 10 mM HEPES, 150 mM NaCl, 5 mM EDTA, 100 mM NaF, 2 mM Na_3_VO_4_, protease inhibitor cocktail, and 1% NP-40. The cell lysates were centrifuged at 14,000 rpm at 4°C for 20 min and then supernatants were collected and stored at −70^°^C.

### Preparation of Virus Lysates From SARS-CoV-2-Infected Culture Supernatants

To determine the cross-reactivity of SARS-CoV-2 N protein-specific monoclonal antibody (clone 1G10C4 mAb) to each clade of SARS-CoV-2, virus lysates were prepared from SARS-CoV-2 S clade-, GR clade-, and GH clade-infected Vero cells or Calu-3 cells. Vero cells or Calu-3 cells (3 × 10^5^ cells) were cultured in six-well plates. The cells were washed with PBS and then each clade in PBS at an MOI of 0.01 was inoculated into each well and then incubated for 1 h in a 5% CO_2_ incubator at 37°C with shaking every 15 min. The cells were washed with PBS and then cultured in 2 ml of DMEM medium containing 2% FBS at 37^°^C in a CO_2_ incubator. After 72 h, cell culture supernatants were harvested and then viral titer was measured using plaque assay. Each clade of SARS-CoV-2-infected culture supernatants was lysed for 30 min with the cell lysis buffer. The virus lysates were centrifuged at 14,000 rpm at 4°C for 20 min and then supernatants were collected and stored at −70^°^C.

### Western Blotting and Immunoprecipitation

Each virus-infected cell lysate or each virus lysate were separated onto a 4–12% Bis–Tris gradient gel (Thermo Fisher Scientific) and then transferred onto a nitrocellulose membrane. The membrane was blocked with PBST containing 5% skim milk and then incubated with SARS-CoV-2 N protein-specific monoclonal antibody (clone 1G10C4 mAb) in PBST at room temperature for 2 h. The membrane was washed three times with PBST and incubated in PBST containing 5% skim milk and anti-HRP-conjugated goat anti-mouse IgG antibody (1:5,000; Jackson ImmunoResearch Laboratories). Immunocomplexes were detected with ECL solution. For the immunoprecipitation assay, SARS-CoV- 2-, MERS- CoV-, or HCoV-OC43-infected Vero cell lysates were incubated with the SARS-CoV-2 N protein-specific monoclonal antibody (clone 1G10C4 mAb) at 4^°^C for 2 h. Immunocomplexes were isolated with Protein A beads (Repligen, Waltham, MA, United States) and analyzed by western blotting with rabbit anti-SARS-CoV-2 N protein polyclonal antibody (Catalog No. 40588-T62; Sino Biological, Vienna, Austria).

### Pull-Down Assay

To investigate the interaction between SARS-CoV-2 Spike CD and each clade of SARS-CoV-2 N protein, we performed pull-down assay. Each virus lysate was incubated with purified control Fc domain or fusion protein containing the SARS-CoV-2 Spike CD and the Fc domain (SARS-CoV-2 Spike CD-Fc) at 4^°^C for 2 h and then complexes were pulled down with Protein A agarose beads. The pull-down complexes were analyzed by western blotting with the SARS-CoV-2 N protein-specific monoclonal antibody (clone 1G10C4 mAb).

### Confocal Images

Vero cells (5 × 10^4^ cells) were cultured on cover glass in 12-well culture plates for 18 h. The cells were washed with PBS and then each clade of SARS-CoV-2 in PBS at an MOI of 0.1 was inoculated into each well and then incubated for 1 h in a 5% CO_2_ incubator at 37°C with shaking every 15 min. After 1 h, the cells were washed with PBS, and then cultured in DMEM containing 2% FBS (1 ml) at 37^°^C in a CO_2_ incubator. After 48 h, the cells were washed with PBS and then fixed with 4% paraformaldehyde and permeabilized with PBS containing 3% BSA and 0.1% Triton X-100 for 30 min. The fixed cells were incubated with the SARS-CoV-2 N protein-specific monoclonal antibody (0.5 μg/well, clone 1G10C4 mAb) for 2 h. After washing with the PBST containing 3% BSA, the cells were incubated with Alexa Fluor 488–conjugated goat anti-mouse IgG (Catalog No. A11001, 1:500 dilution; Thermo Fisher Scientific) for 1 h. After washing with the PBST containing 3% BSA, nuclei were stained with Hoechst 33258 (5 μg/well; Thermo Fisher Scientific). Cells were observed using a Carl Zeiss LSM710 microscope (Carl Zeiss, Oberkochen, Germany).

### Evaluation of the Interaction Between the SARS-CoV-2 Spike CD and N Protein by “Bait and Prey” ELISA

Immunoplates (96-well) were coated with streptavidin (2 μg/well) overnight at 4^°^C and then blocked with PBST containing 3% BSA. Recombinant SARS-CoV-2 N-Bio-His_6_ protein (3 μg/well) was added to each well and plates were incubated for 2 h at room temperature. After washing with PBST, coronavirus CD-human Fc fusion protein (MERS-CoV Spike CD-Fc or SARS-CoV-2 Spike CD-Fc) serially diluted (1:3) in PBST was added to the wells and incubated for 2 h at room temperature. After washing with PBST, goat anti-human IgG Fc antibody conjugated with horseradish peroxidase and the substrate TMB peroxidase was added to each well. The amount of Spike CD-human Fc fusion protein bound to the recombinant SARS-CoV-2 N-Bio-His_6_ protein in the wells was determined colorimetrically.

To determine whether there was competition between the recombinant SARS-CoV-2 N-His_6_ protein and the recombinant SARS-CoV-2 N-Bio-His_6_ protein for interaction with the SARS-CoV-2 Spike CD-Fc, 96-well immunoplates were coated with streptavidin (2 μg/well) and then recombinant SARS-CoV-2 N-Bio-His_6_ protein was added to each well. Serially diluted (1:3) recombinant SARS-CoV-2 N-His_6_ protein was incubated with SARS-CoV-2 Spike CD-Fc (5 μg/well) for 2 h and then added to the wells of the plate. After incubation at room temperature for 2 h, the plates were washed with PBST and then anti-human IgG Fc antibody conjugated with horseradish peroxidase and substrate were added to each well to determine the amount of SARS-CoV-2 Spike CD-Fc protein bound to the recombinant SARS-CoV-2 N-Bio-His_6_ protein in the wells.

### Detection of SARS-CoV-2 in Cell Culture Media by ELISA

Immunoplates (96-well) were coated with SARS-CoV-2 N protein-specific monoclonal antibody (clone 1G10C4 mAb, 5 μg/well) overnight at 4^°^C and then blocked with PBST containing 3% BSA. Virus lysates from each clade of SARS-CoV-2-infected culture supernatants were serially diluted (1:3) in PBST and added to the wells of the plate. After incubation for 2 h at room temperature, recombinant SARS-CoV-2 Spike CD-Fc protein was added and then HRP-conjugated anti-human IgG antibody was added to each well. After developing with the TMB peroxidase substrate, the amount of SARS-CoV-2 N protein in each well was determined by measuring absorbance at 450 nm using a Spectra Max 250 microplate reader.

## Results

### Production and Characterization of the Anti-SARS-CoV-2 N Protein Monoclonal Antibody

**FIGURE 1:**
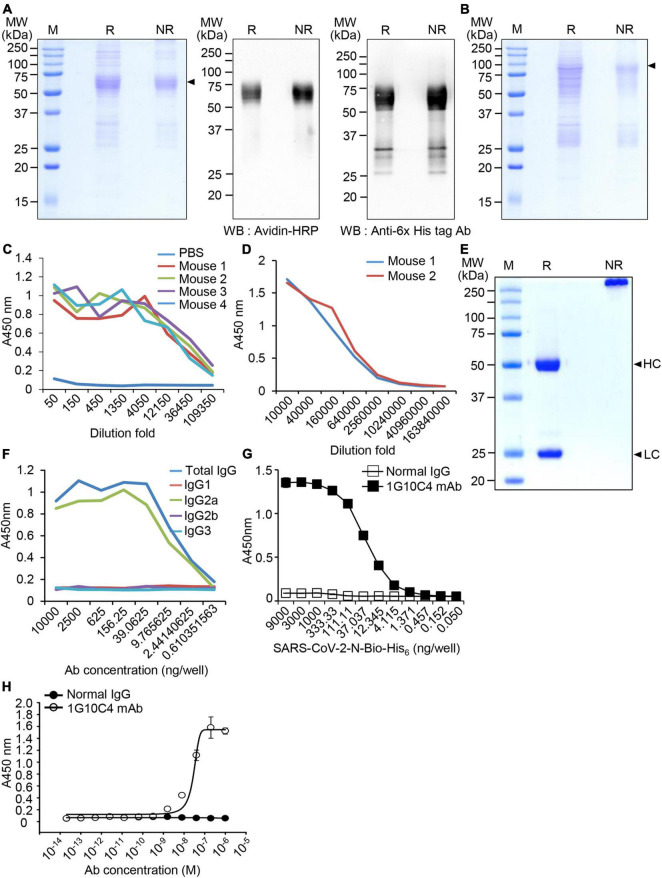
Production and characterization of the anti-SARS-CoV-2 N protein-specific monoclonal antibody. **(A)** Expression of biotin peptide-6 × His-tagged SARS-CoV-2 N protein (SARS-CoV-2 N-Bio-His_6_). Recombinant SARS-CoV-2 N-Bio-His_6_ protein was expressed in ExpiCHO cells and purified from cell culture supernatants using Ni-NTA agarose chromatography. The purified recombinant protein was analyzed by SDS-PAGE (left) and western blotting with peroxidase-conjugated streptavidin (middle) and anti-His-tag antibody (right). Arrowhead, biotin peptide-6 × His-tagged SARS-CoV-2 N protein; R, reducing condition; NR, non-reducing condition. **(B)** Expression of biotin peptide-6 *×* His-tagged MERS-CoV N protein (MERS-CoV N-Bio-His_6_). The purified recombinant protein was analyzed by SDS-PAGE. Arrowhead, biotin peptide-6 × His-tagged MERS-CoV N protein. **(C)** The recombinant SARS-CoV-2 N-Bio-His_6_ protein and CpG-DNA were combined in a DOPE:CHEMS complex and the complex was injected intraperitoneally into BALB/c mice (*n* = 4) three times. ELISA was performed with mouse sera to determine whether recombinant SARS-CoV-2 N-Bio-His_6_ protein-specific antibody was present. **(D)** Ascites were collected from mice injected with cloned hybridoma cells (1G10C4). ELISA was performed with the ascites to determine whether recombinant SARS-CoV-2 N-Bio-His_6_ protein-specific antibody was present. **(E)** The monoclonal antibody was purified from the ascitic fluid using Protein-A column chromatography and analyzed using SDS-PAGE. HC, heavy chain; LC, light chain. **(F)** Subclasses of the monoclonal antibody were identified by ELISA. **(G)** The detection limit of the monoclonal antibody against SARS-CoV-2 N-Bio-His_6_ protein was measured by ELISA. **(H)** Binding of the monoclonal antibody to recombinant SARS-CoV-2 N-Bio-His_6_ protein was measured by ELISA.

To produce monoclonal antibodies against SARS-CoV-2 N protein, biotin peptide-6 × His-tagged SARS-CoV-2 N protein (SARS-CoV-2 N-Bio-His_6_) was expressed in CHO cells in biotinylated form and purified using a Ni-NTA column from cell culture supernatants. The purified recombinant protein was examined by SDS-PAGE and confirmed by western blotting using peroxidase-conjugated streptavidin and an anti-His tag antibody (Figure 1A). We also produced recombinant MERS-CoV N-Bio-His_6_ protein to analyze the specificity of the SARS-CoV-2 N protein-specific monoclonal antibody (Figure 1B). The expected molecular weights for recombinant SARS-CoV-2 N-Bio-His_6_ and recombinant MERS-CoV N-Bio-His_6_ proteins are 50.6 and 49.9 kDa, respectively. However, we found larger protein bands probably because of glycosylation during the expression and secretion of the proteins in CHO cells. We formed a complex containing the purified recombinant SARS-CoV-2 N protein and CpG-DNA co-encapsulated in a liposome (DOPE:CHEMS) and immunized BALB/c mice with this complex. Mouse sera were collected from four immunized mice and production of antibody against recombinant SARS-CoV-2 N protein was confirmed (Figure 1C). Splenocytes were collected from antibody-producing mice and fused with SP2/0, and one hybridoma clone (1G10C4) producing SARS-CoV-2 N protein-specific antibody was selected. The hybridoma cells (1G10C4) were injected into the mouse peritoneal cavity, and the collected ascites were found to contain SARS-CoV-2 N protein-specific monoclonal antibody (Figure 1D), which was purified by Protein A chromatography (Figure 1E). The IgG subclass of the purified monoclonal antibody was IgG2a (Figure 1F). The detection limit of the SARS-CoV-2 N protein-specific monoclonal antibody (clone 1G10C4 mAb) against SARS-CoV-2 N protein was approximately 40 ng/ml (Figure 1G). Binding of the monoclonal antibody to recombinant biotin peptide-6 × His-tagged SARS-CoV-2 N protein was measured by ELISA and the EC50 value was ∼24 nM (Figure 1H).

### Specificity of the Anti-SARS-CoV-2 N Protein Monoclonal Antibody

**FIGURE 2:**
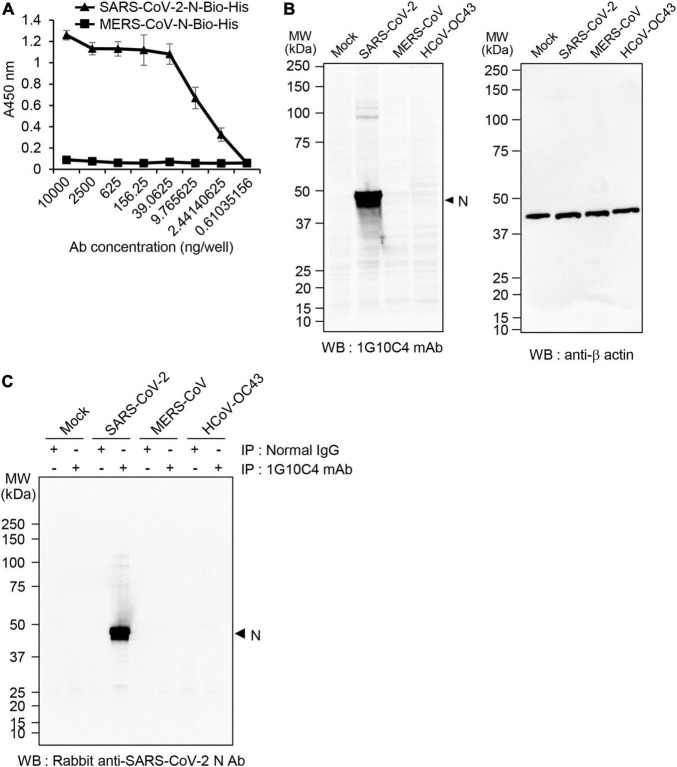
Specificity of the anti-SARS-CoV-2 N protein monoclonal antibody. **(A)** Analysis of monoclonal antibody specificity. Recombinant SARS-CoV-2 N-Bio-His_6_ protein or recombinant MERS-CoV N-Bio-His_6_ protein was captured on streptavidin-coated 96-well immunoplates and then incubated with anti-SARS-CoV-2 N protein monoclonal antibody. Reactivity of the monoclonal antibody to each recombinant protein was determined by ELISA. **(B)** MERS- CoV-, SARS-CoV- 2-, or HCoV-OC43-infected and non-infected Vero cell lysates were immunoblotted with the anti-SARS-CoV-2 N protein monoclonal antibody (clone 1G10C4 mAb). β-Actin was used as the loading control. **(C)** MERS- CoV-, SARS-CoV- 2-, or HCoV-OC43-infected and non-infected Vero cell lysates were immunoprecipitated with normal mouse IgG or the anti-SARS-CoV-2 N protein monoclonal antibody (clone 1G10C4 mAb). The immunocomplexes were subjected to western blot analysis using rabbit anti-SARS-CoV-2 N protein antibody (Catalog No. 40588-T62; Sino Biological).

To determine whether the anti-SARS-CoV-2 N protein-specific monoclonal antibody specifically recognizes the N protein of SARS-CoV-2, ELISA was performed with streptavidin-coated 96-well immunoplates. If we directly coat the recombinant SARS-CoV-2 N-Bio-His_6_ protein on the plates, conformational change of the protein can be induced in the coating buffer condition (pH 9.6). Therefore, we tried to keep the recombinant N protein in a native conformation by coating streptavidin ahead. The monoclonal antibody reacted with the recombinant SARS-CoV-2 N-Bio-His_6_ protein in a concentration-dependent manner (Figure 2A) but did not react with the recombinant MERS-CoV N-Bio-His_6_ protein. To further investigate whether the anti-SARS-CoV-2 N protein monoclonal antibody specifically recognizes the N protein in SARS-CoV-2-infected cells, we performed western blot analysis. The antibody recognized protein with a molecular weight of ∼50 kDa in cell lysates of SARS-CoV-2 (S clade)–infected Vero cells, but not in cell lysates of MERS-CoV- or HCoV-OC43-infected Vero cells (Figure 2B). Immunoprecipitation followed by western blotting with a commercially available antibody that recognizes SARS-CoV-2 N confirmed specific reactivity of the monoclonal antibody with the N protein of SARS-CoV-2 (Figure 2C).

### Anti-SARS-CoV-2 N Protein-Specific Monoclonal Antibody Detects the N Protein of SARS-CoV-2 Clades S, GR, and GH

**FIGURE 3:**
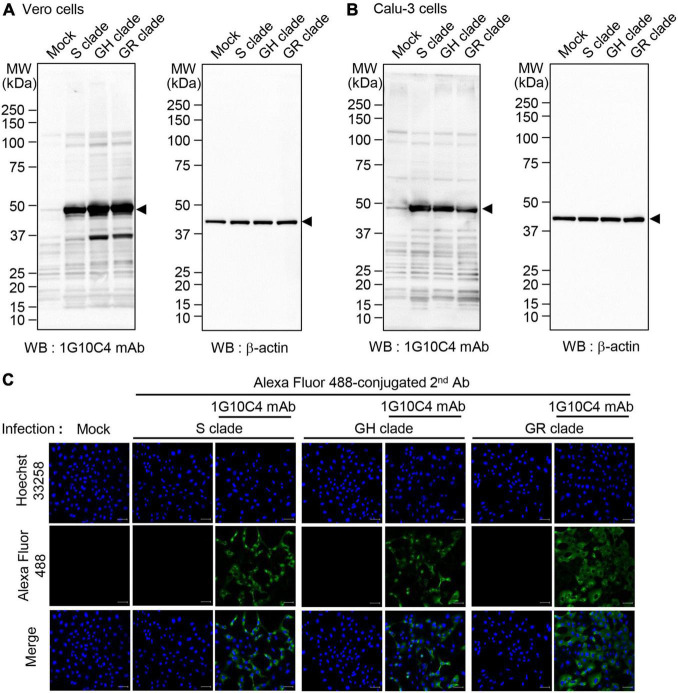
Detection of N protein in cells infected with SARS-CoV-2 S, GH, or GR clade with anti-SARS-CoV-2 N protein-specific monoclonal antibody. **(A,B)** Western blot analyses for the detection of N protein in cells infected with SARS-CoV-2 S, GH, or GR clade. Vero cells **(A)** and Calu-3 cells **(B)** were infected with SARS-CoV-2 S, GH, or GR clade at a MOI of 0.1 for 72 h. Cell lysates were analyzed by western blotting with the anti-SARS-CoV-2 N protein-specific monoclonal antibody (clone 1G10C4 mAb). β-Actin was used as the loading control. **(C)** Confocal microscopy was used to detect N protein in cells infected with SARS-CoV-2 S, GH, or GR clade. Vero cells were cultured on cover glass in 12-well plates and infected with SARS-CoV-2 S, GH, or GR clade at a MOI of 0.1 for 48 h. The infected Vero cells were fixed with 4% paraformaldehyde and permeabilized with 0.1% Triton X-100. The cells were incubated with anti-SARS-CoV-2 N protein-specific monoclonal antibody or normal IgG and then with Alexa Fluor 488–conjugated goat anti-mouse IgG. Nuclei were stained with Hoechst 33258. Images were obtained by confocal microscope. Scale bar, 10 μm.

To investigate whether the anti-SARS-CoV-2 N protein-specific monoclonal antibody recognizes N proteins of different clades of SARS-CoV-2, we infected Vero cells and Calu-3 cells with SARS-CoV-2 clades S, GR, or GH and then performed western blot analysis (Figures 3A,B). The monoclonal antibody recognized N proteins in cell lysates of Vero cells (Figure 3A) and Calu-3 cells (Figure 3B) infected with each of the three clades. We then investigated binding of the monoclonal antibody in SARS-CoV-2-infected cells with confocal microscopy. SARS-CoV-2 (S, GR, or GH clade)–infected and non-infected Vero cells were stained with normal mouse IgG or anti-SARS-CoV-2 N protein-specific monoclonal antibody. Confocal microscopy images showed fluorescence resulting from staining with the anti-SARS-CoV-2 N protein monoclonal antibody for cells infected with each clade. No staining was observed in cells incubated with normal mouse IgG (Figure 3C).

**FIGURE 4:**
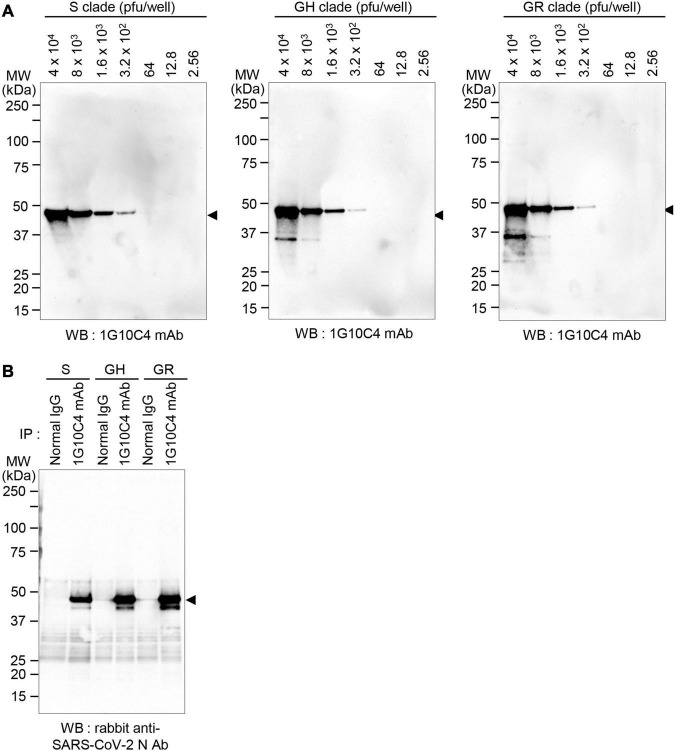
Detection of N protein in SARS-CoV-2 S, GH, or GR clade virus particles with anti-SARS-CoV-2 N protein-specific monoclonal antibody. **(A)** Western blot analyses. Vero cells were infected with SARS-CoV-2 S, GH, or GR clade at a MOI of 0.01 for 72 h and then cell culture supernatants were collected. The cell culture supernatants were lysed with cell lysis buffer and analyzed by western blotting with the anti-SARS-CoV-2 N protein-specific monoclonal antibody (clone 1G10C4 mAb). Virus titers were measured by plaque assay. **(B)** Immunoprecipitation analysis. Vero cells were infected with SARS-CoV-2 S, GH, or GR clade at a MOI of 0.01 for 72 h and then cell culture supernatants were collected. The cell culture supernatants were lysed with cell lysis buffer and immunoprecipitated with normal mouse IgG or the anti-SARS-CoV-2 N protein monoclonal antibody (clone 1G10C4 mAb). The immunocomplexes were subjected to western blot analysis using rabbit anti-SARS-CoV-2 N protein antibody (Catalog No. 40588-T62; Sino Biological).

To further investigate whether the anti-SARS-CoV-2 N protein-specific monoclonal antibody recognizes N proteins in virus particles of the various SARS-CoV-2 clades, viruses in cell culture supernatants of infected cells were lysed with cell lysis buffer, and then western blotting was performed. The monoclonal antibody recognized the N protein in SARS-CoV-2 S clade particles, even at the lower limit of ∼300 pfu (Figure 4A). The monoclonal antibody recognized N proteins in SARS-CoV-2 GH clade particles and SARS-CoV-2 GR clade particles with similar sensitivity. Next, immunoprecipitation assays were performed, and the results confirmed that the anti-SARS-CoV-2 N protein monoclonal antibody reacts with native N proteins in virus particles of each clade (Figure 4B).

These results show that the anti-SARS-CoV-2 N protein-specific monoclonal antibody specifically recognizes N proteins expressed in cells infected with SARS-CoV-2 clades S, GR, or GH and in assembled virus particles of each clade.

### Recombinant SARS-CoV-2 Spike CD Fusion Protein Interacts With the N Protein of SARS-CoV-2 Clades S, GR, and GH Particles

**FIGURE 5:**
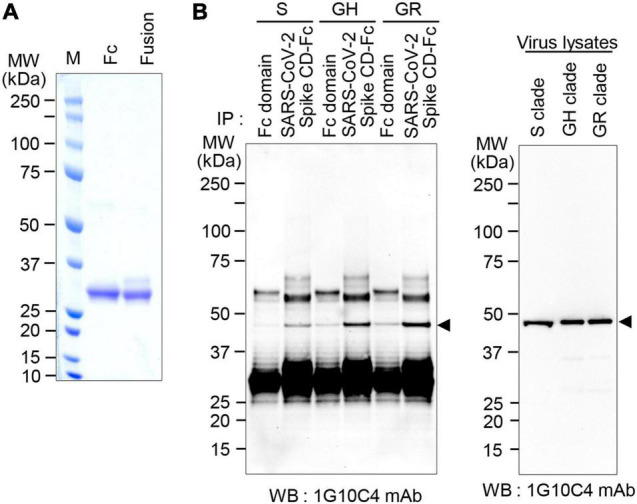
N protein in SARS-CoV-2 S, GH, or GR clade virus particles interact with SARS-CoV-2 Spike CD. **(A)** Expression of SARS-CoV-2 Spike CD-Fc. The recombinant Fc control protein and the SARS-CoV-2 Spike CD-Fc fusion protein were expressed in ExpiCHO cells, purified from cell culture supernatants using Protein A column chromatography and analyzed by SDS-PAGE and Coomassie blue staining. **(B)** Interaction of the N protein in virus particles with SARS-CoV-2 Spike CD. Vero cells were infected with SARS-CoV-2 S, GH, or GR clade at a MOI of 0.01 for 72 h and then cell culture supernatants were collected. Cell culture supernatants were lysed with cell lysis buffer and incubated with Fc or SARS-CoV-2 Spike CD-Fc. Fc-bound proteins were pulled down with Protein A beads and subjected to western blot analysis using the anti-SARS-CoV-2 N protein-specific monoclonal antibody (clone 1G10C4 mAb). Western blot of virus lysates is shown as a control.

Previously, we demonstrated a direct interaction between SARS-CoV-2 Spike CD and SARS-CoV-2 N protein (Park et al., 2021a). To confirm the interaction *in vitro*, we produced and purified control Fc domain and fusion protein containing the SARS-CoV-2 Spike CD and the Fc domain (SARS-CoV-2 Spike CD-Fc; Figure 5A). We incubated the purified SARS-CoV-2 Spike CD-Fc or the Fc control with SARS-CoV-2 clades S, GR, or GH virus lysates and pulled down complexes with Protein A agarose beads. The results showed that N proteins of each clade associated preferentially with SARS-CoV-2 Spike CD-Fc when compared with the Fc control (Figure 5B).

### Recombinant SARS-CoV-2 Spike CD Fusion Protein Binds With the Recombinant SARS-CoV-2 N Fusion Protein

**FIGURE 6:**
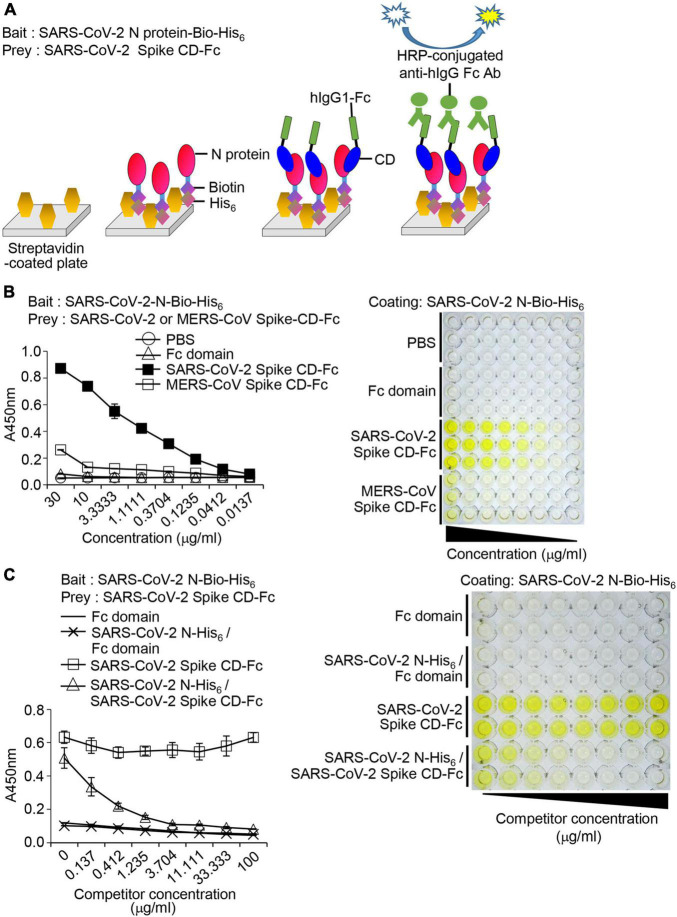
Interaction between SARS-CoV-2 Spike CD-Fc and SARS-CoV-2 N-Bio-His_6_ recombinant proteins. **(A)** Schematic of the bait and prey assay system. **(B)** Immunoplates (96-well) were coated with streptavidin and then recombinant SARS-CoV-2 N-Bio-His_6_ protein was added to each well. After addition of MERS-CoV Spike CD-Fc or SARS-CoV-2 Spike CD-Fc, the amount of CoV Spike CD-human Fc fusion protein bound to recombinant SARS-CoV-2 N-Bio-His_6_ protein in the wells was determined by ELISA. **(C)** Specificity of the interaction between SARS-CoV-2 Spike CD-Fc and SARS-CoV-2 N-Bio-His_6_ recombinant protein. Serially diluted non-biotinylated-recombinant SARS-CoV-2 N-His_6_ protein was incubated with SARS-CoV-2 Spike CD-Fc and then added to the wells containing SARS-CoV-2 N-Bio-His_6_-coated streptavidin. The extent of the competition was measured by ELISA using HRP-conjugated anti-human IgG Fc antibody.

We designed a bait and prey assay system employing streptavidin, SARS-CoV-2 N-Bio-His_6_, SARS-CoV-2 Spike CD-Fc, and anti-human IgG Fc antibody conjugated with HRP (Figure 6A). The ELISA results showed that SARS-CoV-2 Spike CD-Fc bound to SARS-CoV-2 N-Bio-His_6_ in a concentration-dependent manner but the Fc domain and PBS controls did not (Figure 6B). MERS-CoV-2 Spike CD-Fc bound weakly to SARS-CoV-2 N-Bio-His_6_, but only at a high concentration (30 μg/ml). To further investigate the specificity of the interaction between SARS-CoV-2 Spike CD-Fc and SARS-CoV-2 N-Bio-His_6_, competition assays were performed by preincubating with non-biotinylated-recombinant SARS-CoV-2 N-His_6_ protein. The non-biotinylated-recombinant SARS-CoV-2 N-His_6_ protein reduced binding of SARS-CoV-2 Spike CD-Fc with SARS-CoV-2 N-Bio-His_6_ in a concentration-dependent manner (Figure 6C). The Fc domain did not interact with SARS-CoV-2 N-Bio-His_6_ in the presence or absence of non-biotinylated-recombinant SARS-CoV-2 N-His_6_ protein. These results support our previous finding that the SARS-CoV-2 Spike CD and the N protein of SARS-CoV-2 interact specifically and directly and suggests that our bait and prey system using recombinant fusion proteins can be used to quantitatively assess this interaction *in vitro*.

### Detection of SARS-CoV-2 by an ELISA System Based on Interaction Between the Recombinant SARS-CoV-2 Spike CD Fusion Protein and the N Protein of SARS-CoV-2 Particles

**FIGURE 7:**
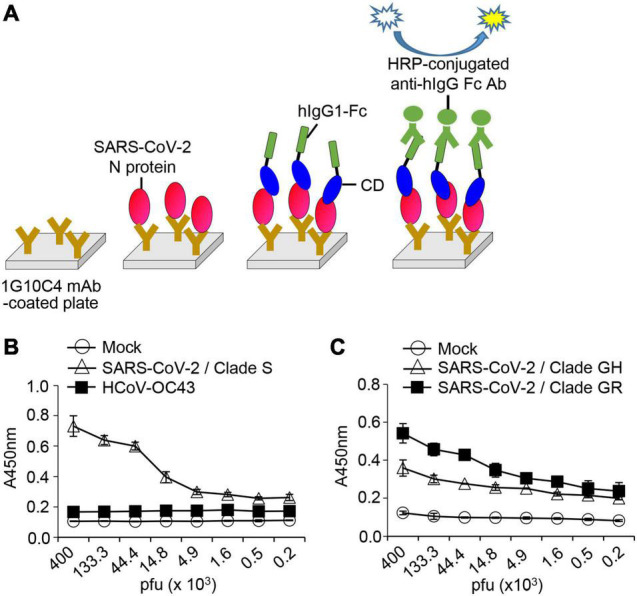
Detection of SARS-CoV-2 in cell culture media using SARS-CoV-2 N protein-specific monoclonal antibody and recombinant SARS-CoV-2 Spike CD-Fc protein. **(A)** Schematic of the ELISA. **(B)** SARS-CoV-2 clade S and HCoV-OC43 and **(C)** SARS-CoV-2 clade GH and clade GR in cell culture supernatants were lysed with cell lysis buffer and serially diluted in PBST. These virus lysates were added into 96-well immunoplates coated with SARS-CoV-2 N protein-specific monoclonal antibody. After incubation for 2 h at room temperature, recombinant SARS-CoV-2 Spike CD-Fc protein was added, and then HRP-conjugated anti-human IgG Fc antibody was added to each well. The amount of SARS-CoV-2 N protein in each well was determined by ELISA.

We designed a detection system for SARS-CoV-2 viruses using the anti-SARS-CoV-2 N protein antibody and SARS-CoV-2 Spike CD-Fc (Figure 7A). N proteins of SARS-CoV-2 particles were captured with the anti-SARS-CoV-2 N protein antibody and then allowed to interact with SARS-CoV-2 Spike CD-Fc. This ELISA system detected virus particles of SARS-CoV-2 clades S, GR, and GH, but not of HCoV-OC43, in a concentration-dependent manner (Figures 7B,C). These results show that the interaction between the viral N protein and SARS-CoV-2 Spike CD Fc fusion protein can be applied in a novel SARS-CoV-2 detection system. Because this system works as expected, the N protein epitope detected by the antibody is most likely not involved in the interaction between the N protein and the Spike CD.

## Discussion

The continuing outbreak of COVID-19 caused by SARS-CoV-2 infection poses a serious threat to public health around the world (WHO, 2021). Therefore, various diagnostic methods have been attempted to rapidly detect SARS-CoV-2 infection, including real-time PCR analysis of viral genes and immunological detection of its S or N proteins. In this study, we proposed a novel approach to detect SARS-CoV-2 that uses a high-affinity anti-SARS-CoV-2 N protein-specific monoclonal antibody and an ELISA-based bait and prey system to target the interaction between SARS-CoV-2 Spike CD and the N protein.

The N protein of SARS-CoV-2 binds to and protects viral RNA and contributes to viral packaging. The protein consists of two structural domains, the N-terminal domain (NTD) and the C-terminal domain (CTD), which bind to the viral genome and facilitate dimerization, respectively (Kang et al., 2020; Zhou R. et al., 2020; Zinzula et al., 2020). Because a large amount of N protein is produced during viral infection, it is used widely for the diagnosis of SARS-CoV-2 (Zhang et al., 2020). In addition, antibody-based detection of the N protein is more sensitive than antibody detection of the S protein (Burbelo et al., 2020).

Several reports have described assays to detect the SARS-CoV-2 N protein using a monoclonal antibody (Mertens et al., 2020; Ciotti et al., 2021; Hodge et al., 2021). Hodge et al. (2021) induced polymerization of two monoclonal antibodies that recognize different epitopes on the N protein to improve its detection. Li et al. developed magnetic nanobead-labeled anti-SARS-CoV-2 N protein monoclonal antibody to induce immunomagnetic enrichment and signal amplification. Rapid and highly sensitive measurements of SARS-CoV-2 N protein have also been performed on microfluidic chips (Li and Lillehoj, 2021).

Previously, we developed a novel strategy to produce antibodies against protein antigens using the antigen and CpG-DNA encapsulated within a DOPE:CHEMS complex as an adjuvant (Kim et al., 2011). In this study, we produced and characterized a monoclonal antibody against the N protein of SARS-CoV-2. We confirmed that the anti-SARS-CoV-2 N protein monoclonal antibody binds specifically to SARS-CoV-2, but not to MERS-CoV or HCoV-OC43, in western blotting and immunoprecipitation. Furthermore, through western blotting and confocal microscopy, we showed that this antibody reacts with N proteins in SARS-CoV-2 clades S, GH, and GR. Because the SARS-CoV-2 N protein-specific monoclonal antibody can recognize N protein in ∼300 pfu of SARS-CoV-2 particles, this antibody could improve diagnostic speed and sensitivity.

Recently, we reported that MERS-CoV and SARS-CoV-2 Spike CDs interact with their respective N proteins in infected cells and that inhibition of this interaction reduced viral replication (Park et al., 2021a). We also suggested that this interaction can be targeted to develop new therapeutics against coronaviruses. In that study, we investigated the interaction between the Spike CD and the N protein by co-immunoprecipitation and competition assays for recombinant Spike CD peptides using virus-infected cell lysates that contained N and S proteins. Therefore, we could not completely exclude the possibility that the interaction was mediated indirectly by other proteins. Thus, in this study, we produced the recombinant fusion proteins SARS-CoV-2 Spike CD-Fc and SARS-CoV-2 N-Bio-His_6_ to demonstrate the direct and specific interaction between these proteins *in vitro* using a newly designed bait and prey ELISA system. Because we showed that the Spike CD-N protein interaction is specific and can be measured quantitatively with our bait and prey ELISA system, this assay could be used to screen for potential inhibitors of the Spike CD-N protein interaction that would block SARS-CoV-2 replication. Further, this ELISA system can be modified to use the anti-SARS-CoV-2 N protein monoclonal antibody that we produced and described in this study. Because this monoclonal antibody is sensitive and specific to the SARS-CoV-2 N protein, it can be used to capture the N protein in virus particles present in test samples, and the SARS-CoV-2 Spike CD-Fc fusion protein can be used to detect captured N protein as evidence of SARS-CoV-2 presence. We used virus particles in cell culture supernatants of SARS-CoV-2-infected cells to confirm that this ELISA system works as predicted. Thus, the anti-SARS-CoV-2 N monoclonal antibody in combination with the recombinant fusion proteins SARS-CoV-2 Spike CD-Fc and SARS-CoV-2 N-Bio-His_6_ can be used for virus detection and development of antivirals against SARS-CoV-2.

As the MERS-CoV and SARS-CoV-2 Spike CD proteins specifically interact with their respective N proteins, the same strategy could be applied for specific detection of MERS-CoV and other coronaviruses, and we will test this possibility in the near future. Recently, we have faced multiple novel coronavirus epidemics and pandemics. Our strategy of screening for drugs that target the Spike CD-N protein interaction can be employed promptly once coronavirus genomic sequences are available for a timely defense against emerging coronaviruses. To further evaluate our strategy practically, it is necessary to determine the clinical sensitivity and specificity and to compare its efficacy with that of current methods.

## Data Availability Statement

The original contributions presented in the study are included in the article/supplementary material, further inquiries can be directed to the corresponding authors.

## Ethics Statement

The animal study was reviewed and approved by the Institutional Animal Care and Use Committee of Hallym University.

## Author Contributions

H-JK and YL conceived the project, designed the experiments, and wrote the manuscript. DK, JK, SP, J-KC, MKi, KB, MKa, SM, and MA carried out the experiments. H-JK, DK, JK, SP, and YL analyzed the data. All authors approved the final version of the article.

## Conflict of Interest

The authors declare that the research was conducted in the absence of any commercial or financial relationships that could be construed as a potential conflict of interest.

## Publisher’s Note

All claims expressed in this article are solely those of the authors and do not necessarily represent those of their affiliated organizations, or those of the publisher, the editors and the reviewers. Any product that may be evaluated in this article, or claim that may be made by its manufacturer, is not guaranteed or endorsed by the publisher.

## References

[B1] Altman J. D. Moss P. A. Goulder P. J. Barouch D. H. McHeyzer-Williams M. G. Bell J. I. (1996). Phenotypic analysis of antigen-specific T lymphocytes. *Science* 274 94–96. 10.1126/science.274.5284.94 8810254

[B2] Brown M. H. Boles K. van der Merwe P. A. Kumar V. Mathew P. A. Barclay A. N. (1998). 2B4, the natural killer and T cell immunoglobulin superfamily surface protein, is a ligand for CD48. *J. Exp. Med.* 188 2083–2090. 10.1084/jem.188.11.2083 9841922 PMC2212392

[B3] Burbelo P. D. Riedo F. X. Morishima C. Rawlings S. Smith D. Das S. (2020). Sensitivity in detection of antibodies to nucleocapsid and spike proteins of severe acute respiratory syndrome coronavirus 2 in patients with coronavirus disease 2019. *J. Infect. Dis.* 222 206–213. 10.1093/infdis/jiaa273 32427334 PMC7313936

[B4] Chu H. Hu B. Huang X. Chai Y. Zhou D. Wang Y. (2021). Host and viral determinants for efficient SARS-CoV-2 infection of the human lung. *Nat. Commun.* 12:134. 10.1038/s41467-020-20457-w 33420022 PMC7794309

[B5] Ciotti M. Maurici M. Pieri M. Andreoni M. Bernardini S. (2021). Performance of a rapid antigen test in the diagnosis of SARS-CoV-2 infection. *J. Med. Virol.* 93 2988–2991. 10.1002/jmv.26830 33527409 PMC8014551

[B6] Ferretti L. Wymant C. Kendall M. Zhao L. Nurtay A. Abeler-Dörner L. (2020). Quantifying SARS-CoV-2 transmission suggests epidemic control with digital contact tracing. *Science* 368:eabb6936. 10.1126/science.abb6936 32234805 PMC7164555

[B7] Hodge C. D. Rosenberg D. J. Grob P. Wilamowski M. Joachimiak A. Hura G. L. (2021). Rigid monoclonal antibodies improve detection of SARS-CoV-2 nucleocapsid protein. *MAbs* 13:1905978. 10.1080/19420862.2021.1905978 33843452 PMC8043170

[B8] Kandeel M. Yamamoto M. Al-Taher A. Watanabe A. Oh-Hashi K. Park B. K. (2020). Small molecule inhibitors of middle east respiratory syndrome coronavirus fusion by targeting cavities on heptad repeat trimers. *Biomol. Ther.* 28 311–319. 10.4062/biomolther.2019.202 32126736 PMC7327142

[B9] Kang S. Yang M. Hong Z. Zhang L. Huang Z. Chen X. (2020). Crystal structure of SARS-CoV-2 nucleocapsid protein RNA binding domain reveals potential unique drug targeting sites. *Acta Pharm. Sin. B.* 10 1228–1238. 10.1016/j.apsb.2020.04.009 32363136 PMC7194921

[B10] Kavithaa K. Paulpandi M. Balachandar V. Ramesh M. Narayanasamy A. (2020). Production and application of polyclonal antibodies against SARS-CoV-2 viral spike protein. Development to rapid, highly sensitive diagnosis kit for early Corona viral detection among the population. *Eur. Rev. Med. Pharmacol. Sci.* 24 10219–10221. 10.26355/eurrev_202010_23244 33090431

[B11] Khailany R. A. Safdar M. Ozaslan M. (2020). Genomic characterization of a novel SARS-CoV-2. *Gene Rep.* 19:100682. 10.1016/j.genrep.2020.100682 32300673 PMC7161481

[B12] Kim D. Maharjan S. Kim J. Park S. Park J. A. Park B. K. (2021). MUC1-C influences cell survival in lung adenocarcinoma Calu-3 cells after SARS-CoV-2 infection. *BMB Rep.* 54 425–430. 10.5483/BMBRep.2021.54.8.018 33832550 PMC8411043

[B13] Kim D. Kwon S. Rhee J. W. Kim K. D. Kim Y. E. Park C. S. (2011). Production of antibodies with peptide-CpG-DNA-liposome complex without carriers. *BMC Immunol.* 12:29. 10.1186/1471-2172-12-29 21592346 PMC3124422

[B14] Lee J. H. Choi M. Jung Y. Lee S. K. Lee C. S. Kim J. (2021). A novel rapid detection for SARS-CoV-2 spike 1 antigens using human angiotensin converting enzyme 2 (ACE2). *Biosens. Bioelectron.* 171:112715. 10.1016/j.bios.2020.112715 33099241 PMC7560266

[B15] Li J. Lillehoj P. B. (2021). Microfluidic magneto immunosensor for rapid, high sensitivity measurements of SARS-CoV-2 nucleocapsid protein in serum. *ACS Sens.* 6 1270–1278. 10.1021/acssensors.0c02561 33629833 PMC7931624

[B16] Maharjan S. Kang M. Kim J. Kim D. Park S. Kim M. (2021). Apoptosis enhances the replication of human coronavirus OC43. *Viruses* 13 2199. 10.3390/v13112199 34835005 PMC8619903

[B17] Mertens P. De Vos N. Martiny D. Jassoy C. Mirazimi A. Cuypers L. (2020). Development and potential usefulness of the COVID-19 Ag respi-strip diagnostic assay in a pandemic context. *Front. Med.* 7:225. 10.3389/fmed.2020.00225 32574326 PMC7227790

[B18] Park B. K. Maharjan S. Lee S. I. Kim J. Bae J. Y. Park M. S. (2019). Generation and characterization of a monoclonal antibody against MERS-CoV targeting the spike protein using a synthetic peptide epitope-CpG-DNA-liposome complex. *BMB Rep.* 52 397–402. 10.5483/BMBRep.2019.52.6.185 30355437 PMC6605520

[B19] Park B. K. Kim J. Park S. Kim D. Kim M. Baek K. (2021a). MERS-CoV and SARS-CoV-2 replication can be inhibited by targeting the interaction between the viral spike protein and the nucleocapsid protein. *Theranostics* 11 3853–3867. 10.7150/thno.55647 33664866 PMC7914343

[B20] Park B. K. Kim D. Park S. Maharjan S. Kim J. Choi J. K. (2021b). Differential signaling and virus production in Calu-3 cells and Vero cells upon SARS-CoV-2 infection. *Biomol. Ther.* 29 273–281. 10.4062/biomolther.2020.226 33504682 PMC8094074

[B21] Schatz P. J. (1993). Use of peptide libraries to map the substrate specificity of a peptide-modifying enzyme: a 13 residue consensus peptide specifies biotinylation in *Escherichia coli*. *Biotechnology* 11 1138–1143. 10.1038/nbt1093-1138 7764094

[B22] WHO (2021). *Coronavirus Disease (COVID-19) Dashboard.* Geneva: World Health Organization.

[B23] Wu Z. McGoogan J. M. (2020). Characteristics of and Important Lessons from the coronavirus disease 2019 (COVID-19) outbreak in China: summary of a report of 72314 cases from the Chinese center for disease control and prevention. *JAMA* 323 1239–1242. 10.1001/jama.2020.2648 32091533

[B24] Zhang L. Zheng B. Gao X. Zhang L. Pan H. Qiao Y. (2020). Development of patient-derived human monoclonal antibodies against nucleocapsid protein of severe acute respiratory syndrome coronavirus 2 for coronavirus disease 2019 diagnosis. *Front. Immunol.* 11:595970. 10.3389/fimmu.2020.595970 33281824 PMC7691652

[B25] Zheng Z. Monteil V. M. Maurer-Stroh S. Yew C. W. Leong C. Mohd-Ismail N. K. (2020). Monoclonal antibodies for the S2 subunit of spike of SARS-CoV-1 cross-react with the newly-emerged SARS-CoV-2. *Euro. Surveill.* 25:2000291. 10.2807/1560-7917.ES.2020.25.28.2000291 32700671 PMC7376845

[B26] Zhou P. Yang X. L. Wang X. G. Hu B. Zhang L. Zhang W. (2020). A pneumonia outbreak associated with a new coronavirus of probable bat origin. *Nature* 579 270–273. 10.1038/s41586-020-2012-7 32015507 PMC7095418

[B27] Zhou R. Zeng R. von Brunn A. Lei J. (2020). Structural characterization of the C-terminal domain of SARS-CoV-2 nucleocapsid protein. *Mol. Biomed.* 1:2. 10.1186/s43556-020-00001-4 34765991 PMC7406681

[B28] Zinzula L. Basquin J. Bohn S. Beck F. Klumpe S. Pfeifer G. (2020). High-resolution structure and biophysical characterization of the nucleocapsid phosphoprotein dimerization domain from the Covid-19 severe acute respiratory syndrome coronavirus 2. *Biochem. Biophys. Res. Commun.* 538 54–62. 10.1016/j.bbrc.2020.09.131 33039147 PMC7532810

